# The Effect of *LINC01296* Expression in Patients with Cancer: A Systematic Review and Meta-Analysis

**DOI:** 10.31557/APJCP.2020.21.8.2189

**Published:** 2020-08

**Authors:** Farzane Saeidi, Kiarash Tanha, Mostafa Davoodabadi Farahani, Ehsan Sohrabi, Yousef Moradi, Pouria Khani

**Affiliations:** 1 *Department of Medical Genetics, School of Medical Sciences, Tarbiat Modares University, Tehran, Iran. *; 2 *Department of Biostatistics, School of Public Health, Iran University of Medical Sciences, Tehran, Iran. *; 3 *Department of Medical Genetics, Faculty of Medicine, Shiraz University of Medical Sciences (SUMS), Shiraz, Iran. *; 4 *Department of Medical Genetics and Molecular Biology, Faculty of Medicine, Iran University of Medical Sciences (IUMS), Tehran, Iran. *; 5 *Department of Epidemiology, School of Public Health, Iran University of Medical Sciences, Tehran, Iran.*; 6 *Social Determinants of Health Research Center, Research Institute for Health Development, Kurdistan University of Medical Sciences, Sanandaj, Iran. *; 7 *Department of Medical Genetics, Faculty of Medicine, Tehran University of Medical Sciences (TUMS), Tehran, Iran. *

**Keywords:** LINC01296, survival, cancer, biomarker

## Abstract

**Background:**

Recently has been suggested that *LINC01296* has an important role in tumor-promoting in different malignancies. We performed first meta-analysis to assess the association between the *LINC01296* expression and clinicopathological criteria and the survival of patients with cancers.

**Methods:**

Relevant articles Identified by PubMed, EMBASE, Web of Science, and Scopus searching between December 2000 and 28 December 2018. Binomial data were evaluated by the odds ratio (OR) as the rapid statistic. The association between overall survival (OS) and the *LINC01296* expression was evaluated using pooling the hazard ratio (HR) with its corresponding 95% confidence interval (CI).

**Results:**

Finally, 9 studies with 720 patients with cancer were included. The expression of *LINC01296* showed a significant positive association with TNM stage (OR = 2.67, 95% CI = 1.83-3.88), tumor stage (OR= 2.22, 95% CI= 1.34-3.66) and lymph node metastasis (OR = 3.07, 95% CI = 2.23-4.21). A shorter OS was significantly associated with the expression of *LINC01296* (HR = 3.95, 95% CI = 2.65-5.25) and lymph node metastasis (HR = 2.39, 95% CI =1.16-3.63). The OS did not show significant association with gender (HR = 0.83, 95% CI = -0.63-2.30) and tumor stage (HR= 2.66, 95% CI= -0.22-5.54).

**Conclusion:**

In conclusion, the results of this meta-analysis suggest that the expression of *LINC01296* might be considered as a potential biomarker in patients with cancer.

## Introduction

More than 90% of the genome of human is transcribed as non-coding RNAs (ncRNAs). This subject suggests that ncRNAs as functional RNA molecules might involve in regulation of gene expression at the transcriptional and post-transcriptional levels in human cells (Ponting and Belgard, 2010; Siegel et al., 2014; Kurozumi et al., 2017). Short ncRNAs (eg microRNAs or miRNAs) and long ncRNAs (or lncRNA) are two broad categories of ncRNAs and in recent years have been revealed to have key functions in various biological processes (i.e., growth, differentiation) especially in cancers (Kurozumi et al., 2017; Monteleone and Lutz, 2017). lncRNAs are a various class of ncRNAs with more than 200 nucleotides in length (Bartonicek et al., 2016). They can be located within introns, between genes or be transcribed as natural antisense of coding genes and play prominent roles in development of various normal cells and diseases progression (Derrien et al., 2012; Kung et al., 2013). Aberrant expression and dysregulation of lncRNAs such as PCGEM1, SPRY4-IT1, PANDAR, and ROR has been shown in malignant cells (Srikantan et al., 2000; Jiang et al., 2017; Liu et al., 2017; Xu et al., 2018). According to the position and transcription direction, lncRNAs are categorized into subgroups including antisense, overlapping, intergenic, intronic, bidirectional, and processed (Bartonicek et al., 2016). Given that understanding of involved non-coding RNAs in cancer pathogenesis could contribute to better treatment of cancer (Xu et al., 2017).

Long intergenic non-protein-coding RNA 1296 (*LINC01296*) is mapped to 14q11.2 and plays an important role in several types of cancers. *LINC01296* has been suggested acts as a tumor-promoting in different malignancies including gastric cancer(Qin et al., 2018), prostate cancer (Wu et al., 2017), colorectal cancer (Qiu and Yan, 2015). Up regulation of *LINC01296* in gastric cancer cells and cell lines resulted to aggravate GC development using the miR-122/MMP-9 axis (Qin et al., 2018). It has been shown *LINC01296* silencing could suppress proliferation of prostate cancer cell and its invasion. Moreover, its expression could be introduced as a biomarker for survival analysis in prostate cancer patients (Wu et al., 2017). Overexpression *LINC01296 *was associated with poor prognosis of patients with colorectal cancer (Qiu and Yan, 2015). Up regulation of *LINC01296* in pancreatic ductal adenocarcinoma tissues and non-small cell lung cancer was related to cell growth and progression, clinical characteristics and poor prognosis (e.g. lymph node metastasis and increasing in TNM stage)(Xu et al., 2019; Yuan et al., 2019).

In this study, we hypothesized that *LINC01296* might inﬂuence the survival of patients with various cancers. There is no meta-analysis has been implemented to assess the correlation between *LINC01296* and the survival of patients with cancers. Hence, we decided to perform this meta-analysis study.

## Materials and Methods

*Study Design*

This article was reported according to recommended guidelines Cochrane library for systematic reviews; the Preferred Reporting Items for Systematic Reviews and Meta-analysis (PRISMA) (Moher et al., 2009a).

*Search strategy*

Identification of relevant articles for this study performed by searches of PubMed, EMBASE, Web of Science, and Scopus between December 2000 and 28 December 2018. The electronic search terms were [*LINC01296* and Cancer (with special MeSH terms for PubMed and Emtree terms for Embase)]. Manual search was performed in reference list and citations of included studies as well as related websites. Two authors (PKH and FS) were cooperated in all stages. Differences were discussed until agreement was reached. It was necessary to contact study author for several times. Country was not limited in this search strategy.

*Inclusion and exclusion criteria*

We included studies that meet the subsequent criteria: (1) case control and cohort studies that evaluate the correlation between the clinicopathological parameters and *LINC01296* in patients with cancer; (2) cancer should be diagnosed according to histopathological evaluation; (3) None of the patients had undergone radiotherapy or chemotherapy treatment before surgery (4) full articles published in English. In this study we excluded: (1) the studies that solely were related to cancer or *LINC01296*; (2) animal study; (3) the study was a review or abstract; (4) Studies whose information was inaccessible.

*Data Extraction and Risk of Bias*

Two authors (PKH and FS) independently reviewed by title, abstract and full text and extracted the data from identified eligible studies. Probable discrepancies between two independent experts were resolved under the supervision of the main investigator (YM). The following data were extracted: the first author’s name first author; year of publication; the patient gender; site of cancer and tumor type; tumor stage; sample size and co-variants; outcome; type of treatment interval; experimental techniques used. Finally, we using the Newcastle-Ottawa Scale (NOS) assessed the methodological quality of included studies (Wells, 2001).

*Statistical analysis*

Binomial data were evaluated by the odds ratio (OR) as the rapid statistic. The correlation between *LINC01296* and survival of cancer was evaluated by combining the HR with its corresponding 95% CI. The heterogeneity of studies examined by I2 statistic and the chi-squared based Q-statistic test. The fixed effect models were used to estimate the pooled effect sizes across studies. Determination of all the P-values was performed using a 2-sided test. Software for all statistical analyses was STATA software version 13.

*Ethics statement*

Consent to participate was unnecessary.

**Figure 1:**
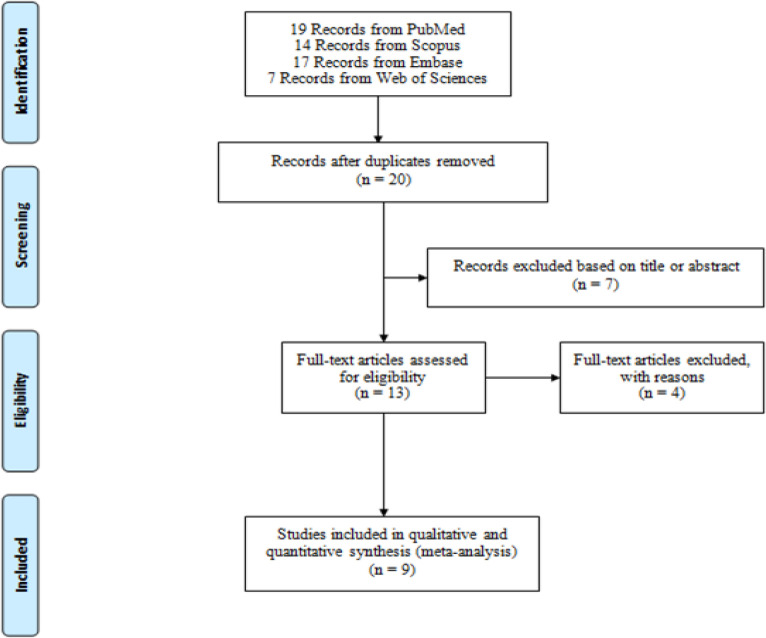
PRISMA Flow Diagram of Study Identification

**Figure 2:**
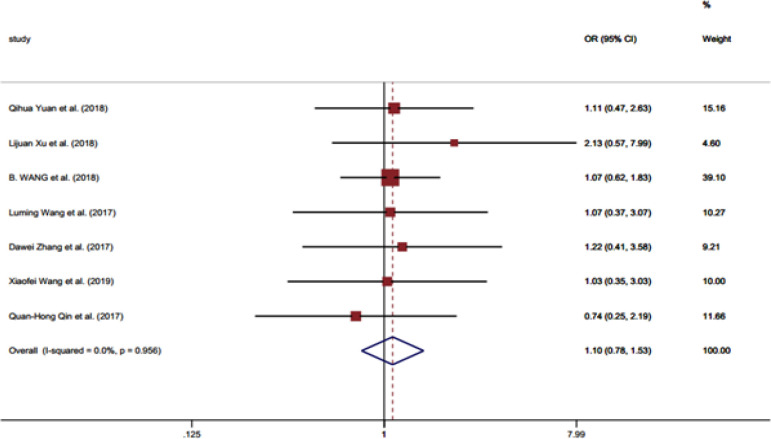
Forest Plot Illustrating the Association between the *LINC01296* Expression and Gender of Patients

**Table 1:** Features of the Included Studies

First Author	Year of publication	Gender ( Male / Female)	Type of cancer	TNM stage	Sample Size	Outcome	Lymph node metastasis (Present / Absent)	Tumor Stage	NOS
Yuan, Q., et al.	2018	47 / 38	Pancreatic ductal adenocarcinoma	NA	85	OS	53 / 32	T1-4	7
Xu, L., et al.	2018	26 / 14	NSCLC	I-IV	40	OS*	20 / 20	NA	7
Wang, B., et al.	2018	134 / 87	Esophageal squamous cell carcinoma	I-IV	221	OS	83 / 138	NA	7
Wang, L., et al.	2017	60 / 18	Esophageal squamous cell carcinoma	I-IV	78	OS*	27 / 51	T1-4	7
Zhang, D., et al.	2017	32 / 25	Cholangiocarcinoma	I-IV	57	OS*	28 / 29	NA	7
Jiang, M., et al.	2018	55 (Female)	Breast cancer	I-III	55	OS	36 / 19	NA	7
Wang, X., et al.	2019	30 / 24	Urothelial carcinoma of the bladder	NA	54	OS	18 / 36	NA	7
Wu, J., et al.	2017	73 (Male)	Prostate cancer	NA	70	OS	38 / 32	T2-4	7
Qin, Q.H., et al.	2017	33 / 27	Gastric cancer	I-III	60	OS*	48 / 12	T1-3	7

OS, overall survival; OS*, no data of OS could be extracted; NOS, Newcastle-Ottawa Scale; NA, not available; NSCLC, Non‐small cell lung cancer

**Figure 3:**
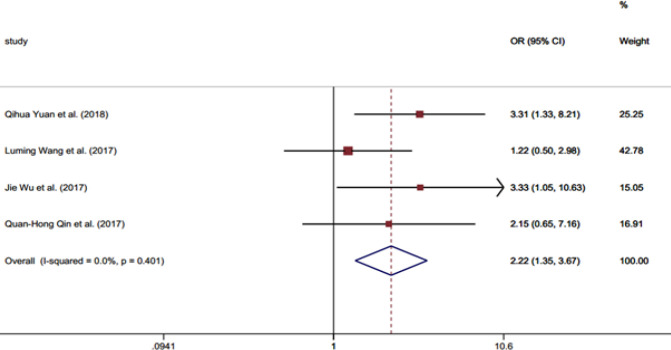
Forest Plot Illustrating the Association between the *LINC01296 *Expression and Tumor Stage

**Table 2:** The Association between *LINC01296* and Clinicopathological Criteria AND Univariate Analysis of Prognostic Factors for Overall Survival

	Correlation test			Heterogeneity	
	OR	95% CI	*P*-value	I2 (%)	*P*-value
Gender	1.09	0.78-1.53	0.592	0	0.956
Age	1.31	0.96-1.8	0.086	0	0.556
TNM stage	2.67	1.83-3.88	0	0	0.914
Tumor stage	2.22	1.34-3.66	0.002	0	0.401
Lymph node metastasis	3.07	2.23-4.21	0	45.6	0.065
Gender	0.83	-2.93	0.265	0	0.998
Tumor stage	2.66	-5.76	0.071	65.7	0.054
Lymph node metastasis	2.39	1.16-3.63	0	0	0.801
LINC01296 expression (High vs Low)	3.95	2.65-5.25	0	26.1	0.248

**Figure 4:**
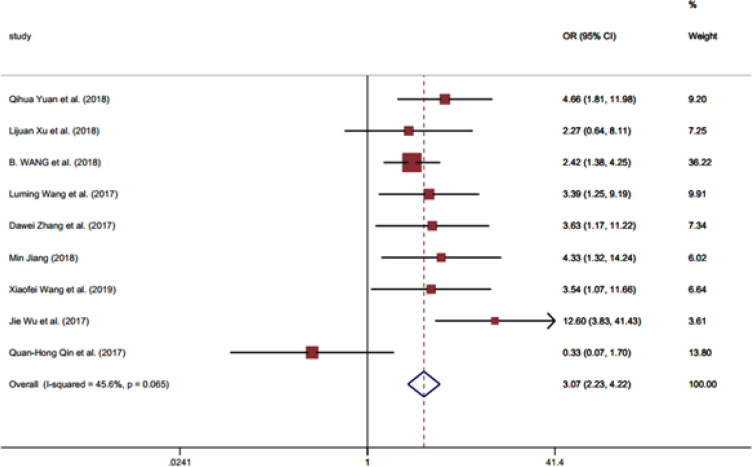
Forest Plot Illustrating the Association between the *LINC01296* Expression and Lymph Node Metastasis

## Results

*Study characteristics*

Figure 1 show the PRISMA flow diagram (Moher et al., 2009b) of study identification of relevant studies. Fifty-seven studies were found in the preliminary search. After a comprehensive evaluation comprises remove of duplicates, exclusion based on title or abstract and exclusion based on some reasons such as inaccessibility to their data, finally, 9 studies with 720 patients with cancer were included in this meta-analysis. Pancreatic ductal adenocarcinoma (PDAC), non-small cell lung cancer, Esophageal squamous cell carcinoma (ESCC), Cholangiocarcinoma, breast cancer, urothelial carcinoma of the bladder, prostate cancer, gastric cancer was studied in the original studies. The characteristics of the included studies have been shown in Table 1. The data of Overall Survival (OS) extracted from Only 5 studies.

*Results of the meta-analysis*

Table 2 shows the associations between the clinicopathological criteria and the *LINC01296* expression. No significant association was identified between the *LINC01296* expression and gender (OR = 1.10, 95% CI = 0.78-1.53) (Figure 2 and Table 2). Moreover, the *LINC01296* expression was not related to the patients age (OR = 1.31, 95% CI =0.96-1.8) (Table 2). Conversely, the expression of *LINC01296* showed a significant positive association with TNM stage (OR = 2.67, 95% CI = 1.83-3.88) (Table 2), tumor stage (OR= 2.22, 95% CI= 1.34-3.66) (Table 2 and Figure 3) and lymph node metastasis (invasion) (OR = 3.07, 95% CI = 2.23-4.21) (Figure 4). Additionally, a shorter OS (overall survival) in patients was significantly associated with the expression of this RNA (HR = 3.95, 95% CI = 2.65-5.25) and lymph node metastasis (HR = 2.39, 95% CI =1.16-3.63) (Table 2). The OS did not show significant association with gender (HR = 0.83, 95% CI = -0.63-2.30) and tumor stage (HR= 2.66, 95% CI= -0.22-5.54) (Table 2).

*Meta Regression*

Meta regression was used to explore the sources of heterogeneity among studies, including age and gender. The results of this this analysis showed that the associations are not related to age (B= -0.00043, P=0.345, 95% CI -0.007, 0.0010) and gender (B= 0.0380, P=0.478, 95% CI -0.046, 0.022).

*Publication Bias Assessment*

The results of Egger’s test show no significant bias occurred in the publication of the results (Egger’s test = 1.01 SE: 0.221, P = 0.443).

## Discussion

Recently, several studies have demonstrated abnormal expression of *LINC01296* has been associated with several malignancies (Jiang et al., 2018). Therefore, based on information provided by several studies, we performed the first meta-analysis to assess the correlation between the *LINC01296* expression and prognosis based on patients clinicopathological parameters in cancers. The results of our meta-analysis demonstrated that the expression of *LINC01296* was significantly related with lymph node metastasis, TNM stage and Tumor stage. Moreover, prognostic factors univariate analysis for OS in this study, showed a significant association between OS of patients with cancer and the *LINC01296* expression and lymph node metastasis. Since, patients with high expression of *LINC01296* compared with those had a low level of the expression, had a poor prognosis, the expression of *LINC01296* might be considered as a potential biomarker in patients with cancer.

In a study, Anna Katharina Seitz et al.,(Seitz et al., 2017) identified aberrant expression of lncRNAs in 8 normal and 72 breast cancer samples using RNA-sequencing. Among identified 89 lncRNAs that were significantly dysregulated, in vitro *LINC01296* silencing reduced cell viability and migration. Moreover, they demonstrated that both LINC00958 and *LINC01296* localized to both the nucleus and the cytoplasm and knock-down of them altered the expression of genes related to cell death/survival and cellular growth/proliferation pathways. According to these findings, they suggested that *LINC01296* act as oncogenes by stimulating proliferation and the metastatic process in breast cancer cells (Seitz et al., 2017). XIN YU et al., assessed the levels of *LINC01296* expression in osteosarcoma cells in comparison to normal cells (Yu et al., 2018). Their findings revealed, the up regulation of *LINC01296* was closely associated with the poor survival of osteosarcoma subjects. *LINC01296* acts as an oncogenic factor which is able to increase the proliferation, invasion, and migration of osteosarcoma cells. Moreover, the expression of Cyclin D1 was positively correlated with the *LINC01296* expression in osteosarcoma cells so that *LINC01296* knockdown could lead to down regulation of cyclin D1 (Yu et al., 2018). It has been shown that Overexpression of *LINC01296* is one of very important players in the progression of CRC(Qiu and Yan, 2015). In a Bing Liu et al., (Liu et al., 2018), shown that *LINC01296* correlated with poor clinical prognosis in patients and the malignancy of CRC cell lines. Alteration of the *LINC01296* expression impresses proliferation and metastasis of CRC cell and cause in vitro chemoresistance to 5-fluorouracil (5-FU). *LINC01296* as a miR-26a direct target through *LINC01296*/miR-26a/GALNT3 axis encourage CRC malignancy using regulating O-glycosylated MUC1 by PI3K/AKT pathway. Upregulation of *LINC01296* stimulates the in vivo tumorigenesis, liver metastasis and chemoresistance of CRC cell lines(Liu et al., 2018). Upregulation of *LINC01296* was significantly seen in some another cancers such as gastric cancer, prostate cancer, urothelial carcinoma of the bladder and esophageal squamous cell carcinoma that in all of them correlated with lymph node metastasis, advanced TNM stage, and shorter overall survival(Wu et al., 2017; Qin et al., 2018; Wang et al., 2018; Wang et al., 2019).

*LINC01296* knockdown using siRNAs could be related to inducing of cell apoptosis. *LINC01296* could suppress cell apoptosis via targeting the Bcl-2/caspase-3 pathway(Yuan et al., 2019). Bcl-2 is known as a central regulator of cell life and death and caspase-3 acts as an effector caspase which has a crucial role in cell apoptosis process (Evan and Littlewood, 1998; Mantena et al., 2006). In an epithelial-mesenchymal transition (EMT) process, a polarized epithelial cell by multiple biochemical changes accepts a mesenchymal cell phenotype. Gained features of this phenotype comprise enhanced capability of migratory, invasiveness, higher apoptosis resistance, and significantly increased ECM components production(Kalluri and Neilson, 2003). The loss of epithelial proteins such as E-cadherin and higher expression of nonepithelial markers such as N-cadherin and Vimentin are of the important features of EMT(Dong et al., 2012). Knockdown of INC01296 by reversing Snail-mediated EMT decrease the capacities of invasiveness and migration. Taken together, these findings, the axis of *LINC01296*/Snail/ E-cadherin may have an important role in facilitating metastatic properties of pancreatic ductal adenocarcinoma(Yuan et al., 2019).

At the end of, some limitations of this meta-analysis comprise of following: 1) only 5 studies provide the survival data and only 1 study could provide the disease-free survival data. 2) The sample size collected for overall survival analysis in this study was small that this subject does not provide statistical power to make a significant assessment.3) Also, implementation of subgroup analysis in one specific cancer or based on the ethnicity due to inadequate data was not possible. 4) Because of not doing an in vitro experiment, therefore, more studies for results confirmation of this meta-analysis are needed. In conclusion, this meta-analysis suggests that *LINC01296* may be a diagnostic biomarker for shorter OS and metastasis.

In conclusion, cancer is one of very important public health problems which is associated with high rate mortality and mobility in patients with this disease. Finding, designing and developing new diagnostic platforms could contribute to increase survival rate of cancer patients. In this regards, a variety of biomarkers have been introduced by pre-clinical studies. Taken together, the results of this meta-analysis suggest that the expression of *LINC01296* might be considered as a potential biomarker in patients with cancer.

## Abbreviations

ncRNAs: non-coding RNAs

microRNAs: Short ncRNAs

lncRNA: long ncRNAs

PCGEM1: prostate cancer gene expression marker 1

SPRY4-IT1: SPRY4 Intronic Transcript 1

PANDA: P21-associated ncRNA DNA damage –activated

RoR: lincRNA-regulation of reprogramming

*LINC01296*: Long intergenic non-protein-coding RNA 1296

TNM: Tumour, Node, Metastasis

PRISMA: The Preferred Reporting Items for Systematic Reviews and Meta-analysis

MeSH: Medical Subject Headings

OS: overall survival
